# An initiative to implement a triage and referral system to make exercise and rehabilitation referrals standard of care in oncology

**DOI:** 10.1007/s00520-024-08457-8

**Published:** 2024-04-02

**Authors:** Kathryn H. Schmitz, Andrew Chongaway, Anwaar Saeed, Toni Fontana, Kelley Wood, Susan Gibson, Jennifer Trilk, Prajakta Adsul, Stephen Baker

**Affiliations:** 1 Division of Hematology and Oncology, School of Medicine, UPMC Hillman Cancer Center, University of Pittsburgh, 580 S. Aiken Ave, Suite 610, Pittsburgh, PA 15232 USA; 2 ReVital Cancer Rehabilitation, Select Medical, Mechanicsburg, PA USA; 3 Hillman Cancer Center, University of Pittsburgh Medical Center, Pittsburgh, PA USA; 4 Department of Biomedical Sciences, University of South Carolina School of Medicine, Greenville, SC USA; 5 Division of Epidemiology, Biostatistics, and Preventive Medicine, Department of Internal Medicine, School of Medicine, University of New Mexico, Albuquerque, NM USA; 6 Comprehensive Cancer Center, Cancer Control and Population Sciences Research Program, University of New Mexico, Albuquerque, NM USA

**Keywords:** Exercise, Rehabilitation, Cancer patients

## Abstract

**Background:**

Clinical guidelines suggest that patients should be referred to exercise while undergoing cancer treatment. Oncology clinicians report being supportive of exercise referrals but not having the time to make referrals. Toward the goal of making exercise referrals standard of care, we implemented and evaluated a novel clinical workflow.

**Methods:**

For this QI project, a rehabilitation navigator was inserted in chemotherapy infusion clinics. Patients were offered a validated electronic triage survey. Exercise or rehabilitation recommendations were communicated to patients during a brief counseling visit by the rehabilitation navigator. The implementation approach was guided by the EPIS framework. Acceptability and feasibility were assessed.

**Results:**

Initial meetings with nursing and cancer center leadership ensured buy-in (exploration). The education of medical assistants contributed to the adoption of the triage process (preparation). Audit and feedback ensured leadership was aware of medical assistants’ performance (implementation). 100% of medical assistants participated in implementing the triage tool. A total of 587 patients visited the infusion clinics during the 6-month period when this QI project was conducted. Of these, 501 (85.3%) were offered the triage survey and 391 (78%) completed the survey (acceptability). A total of 176 (45%) of triaged patients accepted a referral to exercise or rehabilitation interventions (feasibility).

**Conclusions:**

Implementation of a validated triage tool by medical assistants and brief counseling by a rehabilitation navigator resulted in 45% of infusion patients accepting a referral to exercise or rehabilitation. The triage process showed promise for making exercise referrals standard of care for patients undergoing cancer treatment.

## Introduction

ASCO guidelines for Exercise, Diet, and Weight Management During Cancer Treatment state that clinicians *should* refer their patients to exercise during chemotherapy to address common symptoms and side effects [1]. The benefits of exercise for people living with and beyond cancer are well described and include improvements in physical function, body composition, fatigue, anxiety, depression, sleep, bone health, and quality of life [1–3]. However, published evidence, also from ASCO, suggests that only 15% of patients recall being referred to exercise by their oncologist [4]. People living with and beyond cancer are less likely to be active than their healthy counterparts and published evidence suggests that advice from an oncology clinician results in more physical activity [5].

We know that exercise has benefits when patients participate in exercise oncology programming. What we know less about is how to make referrals to exercise oncology programming. Taken together, new methods are needed for implementing exercise referrals. One complication of this is the recognition that patients vary as to the level of care needed to successfully participate in appropriate exercise programming [6]. Some patients could exercise on their own, some have sufficient impairments that professional guidance would be advisable, and for yet others, there are documentable impairments for which outpatient rehabilitation would be the best course of action [7]. A system is needed to triage patients that can be broadly implemented in oncology care. This is a cancer care delivery problem that can be addressed through research [8].

The Exercise in Cancer Evaluation and Decision Support (EXCEEDS) triage tool was developed to support patients and clinician’s decision-making to participate in or refer to appropriate exercise oncology or rehabilitation services [9]. The tool has been validated using a Delphi process [9] including high satisfaction among oncology clinicians [10]. We undertook a quality improvement (QI) project to make triage and referral to exercise oncology standard of care, by embedding the EXCEEDS tool into clinical care. Goals of the QI initiative were to evaluate whether integrating a rehabilitation navigator and applying the EXCEEDS tool would be acceptable and feasible from the perspective of patients and clinical staff, as well as to assess the extent to which this approach would improve the proportion of patients referred to appropriate exercise or rehabilitation programming. The project was guided by the EPIS framework [11] (Exploration, Preparation, Implementation, and Sustainment) and prior efforts to map exercise oncology implementation strategies onto a compendium of implementation strategies [12].

## Methods

This project was reviewed by the QI committee of the University of Pittsburgh Medical Center (UPMC) and deemed not to be human subjects research, but to be a QI initiative, according to review of the common rule (45 CFR 46.102(d)). SQUIRE 2.0 guidelines were followed [13]. So that we could build on the learnings of this QI project, we aligned the methods and measures used with the approaches promoted by the science of implementation, especially as noted in the cancer prevention and control field [14]. Through this initiative, our goals were to generate practice-based evidence to inform future research and practice-changing activities.

### Implementation framework

The EPIS framework guided this QI initiative. The EPIS framework highlights key phases that guide and describe the implementation process and clarifies common and unique factors within and across levels of system and organizational context across phases, factors that bridge the variety of contexts specific to implementation, and the nature of the innovation being implemented, as well as the role of the innovators [11]. There are four phases to the EPIS Framework: Exploration, Preparation, Implementation, and Sustainment. In this project, we accomplished three of these phases; sustainment remains to be studied. After 5 months, we expanded our program to the 3rd floor of the Hillman Cancer Center. All implementation strategies were repeated for this expansion.

### Triage tool

We chose to use the EXCEEDS tool [9] for our triage and referral program. EXCEEDS has 23 questions, in 3 sections, including a section regarding difficulty completing daily activities (positive responses lead to rehabilitation services referrals); a section about recent falls, recent cancer treatments, and recent symptoms (e.g., fatigue, neuropathy, memory, dizziness, nausea, lymphedema), and a section about catheters, current exercise, and confidence with exercise. It was developed with the help of a multidisciplinary team of experts and has been documented to be acceptable to oncology clinicians [10]. The EXCEEDS tool is intended to result in referral to four possible levels of intervention: unsupervised exercise, supervised cancer-specific community-based exercise, clinically supervised exercise, and cancer rehabilitation. EXCEEDS was designed to be adaptable to the needs of local systems. As such, based on available programming at our site, we collapsed into three levels of intervention: community-based exercise oncology programming, clinically supervised exercise, and cancer rehabilitation. The instrument was loaded into a RedCAP® database and delivered to patients on a computer tablet.

### Flow of clinical activities

A rehabilitation navigator identified patients coming in for their second chemotherapy infusion visit at Hillman Cancer Center. The clinical encounters reported on herein occurred between March 21 and October 6, 2023. A list of patients was provided to the medical assistants. At the point of seating the patient in their chemotherapy chair, the medical assistants provided an iPad to the patient and asked that they complete the survey, which was provided in RedCAP® [15], [16]. It took between 2 and 5 min per patient for medical assistants to complete the task of providing the iPad and explaining the survey. Upon completion, the scored results became immediately available to the rehabilitation navigator, who approached the patient for a brief counseling session to discuss the results and provide the referral. These counseling sessions were usually 5 to 10 min long but could be as long as 90 min. Patients had the option to accept or deny any recommendation made.

#### Program staff

The Moving Through Cancer Triage Program staff includes a licensed physical therapist with specialty training in Exercise Oncology (the rehabilitation navigator) and five Medical Assistants, who provided the iPads to the patients at the start of the second infusion visit.

#### Programs offered

When a patient was identified as having the symptom and disease profile consistent with referral to community-based exercise programming (e.g., minimal symptoms or comorbidities), we offered those who were UPMC health plan members the option to connect with the UPMC Prescription for Wellness (https://www.upmcmyhealthmatters.com/prescription-for-wellness-for-upmc-health-plan-members/) a customized care management system for healthy lifestyle changes. Other options offered included local cancer exercise offerings (Cancer Bridges: https://cancerbridges.org/), or a link to online programming available through the Moving Through Cancer Directory (https://www.exerciseismedicine.org/eim-in-action/moving-through-cancer/). After 4 months during which acceptance of these options was close to zero, we began to offer these patients the option for a few sessions of clinically supervised exercise with the rehabilitation navigator. These sessions provided instruction that could be carried out within unsupervised home-based exercise sessions. There are ongoing and completed exercise oncology interventions that have offered brief in person counseling that have documented benefits [17–19]. Patients had the option to refuse this offering as well.

When patients had sufficient symptoms to warrant more attention, but insufficient for referral to outpatient rehabilitation, the rehabilitation navigator offered clinically supervised exercise sessions. These sessions could occur prior to the infusion visit or at a separate time, and could occur chair side, in the chemotherapy infusion center, in the 200 square-foot gym on the second floor of Hillman Cancer Center, or virtually.

If a patient identified sufficient impairments that the EXCEEDS triage tool suggested referral to outpatient rehabilitation, the rehabilitation navigator offered this referral. If the referral was accepted by the patient, the rehabilitation navigator facilitated the referral by providing supporting materials to the appropriate nursing staff. If patients refused the referral, they were offered clinically supervised exercise sessions with the rehabilitation navigator.

### Statistical analysis

All analyses were conducted in R (version 4.3.2). Descriptive statistics were developed for tables. Acceptability was defined as the percentage of patients who were willing to engage in the triage process. Feasibility was defined as the percentage of triaged patients who accepted a referral. Our a priori threshold for establishing feasibility was 30% (double the published background rate) [4]. Differences between those who accepted the triage process and those who did not were examined using *t*-tests and chi-square tests. Differences in exercise confidence across EXCEEDS triage tool levels were examined using a chi-square test.

## Results

### Implementation results

The exploration phase of implementation focused on building the coalition for this program with key administrators, decision makers, implementers, and leadership champions. In this setting, iterative conversations with staff and leadership led to meetings with the cancer center director, the CEO of the cancer center, the Division Chief of Hematology Oncology, the Senior Director of Hillman Operations, 2nd floor Hillman Nursing Unit Director, Nursing Lead for Treatment Side, 2nd floor Hillman, and the managers for the Medical Assistants and Collaborative Nurses on the 2nd floor of Hillman. Similar meetings occurred when we expanded the program to the 3rd floor clinic. This phase of implementation took approximately 3 months. In the preparation phase, we made presentations in the settings of medical oncology grand rounds and during multiple meetings of nursing leadership and staff. We obtained formal commitment for the project from senior nursing staff. We developed educational training materials that were shared at all meetings and conducted numerous educational outreach visits within the infusion clinic. We further gained the buy-in of nursing staff by demonstrating adaptability to their workflow. We were interested in finding a time during the 2nd infusion visit encounter that had the lowest likelihood of interfering with the flow of clinic activities. The champions chose the interactions of medical assistants with patients as they are being positioned in the chemotherapy chair as the best possible time point. This phase of implementation lasted approximately 6 weeks.

For our implementation phase, we continued regular meetings with appropriate nursing staff to report on our progress. An audit and feedback process with our nursing leadership champions led to increased likelihood of medical assistants complying with the new clinical workflow. The principal investigator (an exercise physiologist) met with the director of operations, who then conveyed the audit and feedback information to nursing leadership. The first occasion of audit and feedback was a phone call discussion; two subsequent occasions were email exchanges. We intervened with medical assistants in another educational meeting when there was a drop in implementation. At present, all 5 participating medical assistants are regularly getting the iPad into the hands of patients at the second infusion visit. The intervention was delivered with fidelity to the planned implementation of this triage and referral process with 100% of medical assistants complying with the necessary actions over the entire 6 month observation period reported herein.

**Fig. 1:**
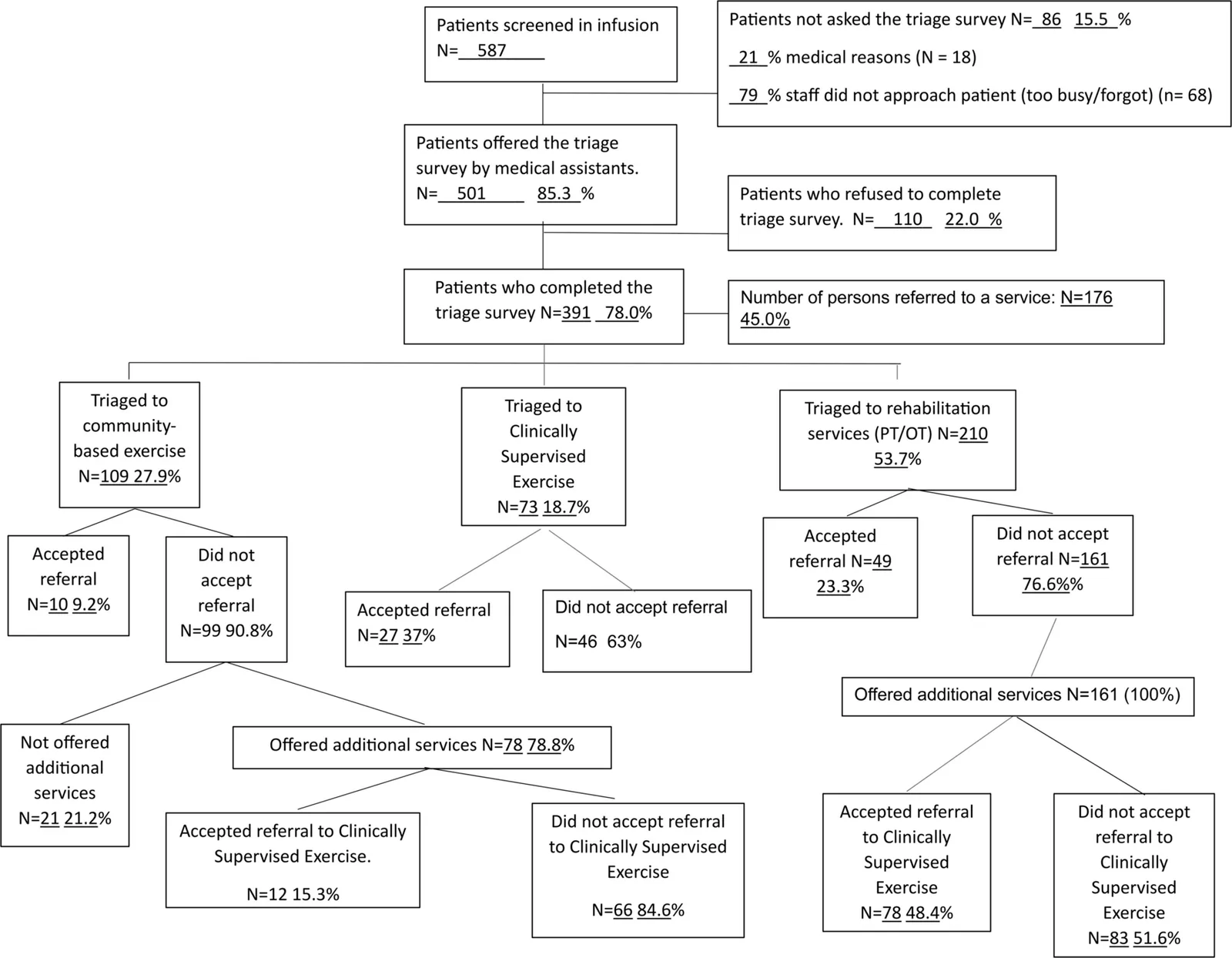
Flow of participants

Figure 1 shows the flow of participants through the triage and referral process. A total of 587 patients were seen for a second infusion visit during the time period of this QI project. Of these, 501 (85.3%) were offered the triage survey by medical assistants. Of these, 78% accepted the triage survey. A total of 391 patients completed the survey and, of these, 176 (45%) were connected to an exercise or rehabilitation intervention (feasibility). The proportion of patients for whom responses to the EXCEEDS triage survey suggested that a community-based exercise intervention was appropriate was 27.9%. Of these, 9.2% accepted the referral. Given the low uptake of this referral, we added the option of a consultation with the rehabilitation navigator after a few months. Of the 78 patients offered this option, 12 (15%) accepted the referral. The proportion of patients triaged to clinical exercise supervision was 18.7%. Of these patients, 37% agreed to the referral. The proportion of triaged patients for whom responses to the EXCEEDS triage tool suggested that it would be appropriate to refer to cancer rehabilitation was 53.7%. Of these, 23.3% accepted this referral. Of those who did not accept the referral to cancer rehabilitation, we offered clinical supervised exercise. Of the 161 offered this option, 78 or 48.4% accepted the referral.

For the remaining 127 patients, a total of 173 total consultations and full exercise sessions were carried out. Most of the patients received 1–3 exercise sessions or consultations (*N* = 108). Nine patients received between 4 and 6 sessions.

Table 1 presents a description of the overall patients approached for the triage and referral system, as well as those who agreed versus those who refused the triage instrument. There were no significant statistical differences between the patients who accepted versus those who did not accept the triage instrument.

**Table 1:** Description of patients

	Overall mean (SD) or *N* (%) , (*N* = 501)	Said yes to triage survey , (*N* = 393)	Said no to triage survey , (*N* = 108)	*P*-value
Age, years	64.96 (12.69)	64.73 (12.69)	65.88 (12.69)	0.42
Race				0.24
Non-Hispanic White	462 (93.33%)	365 (94.07%)	97 (90.65%)	
Black	30 (6.06%)	20 (5.15%)	10 (9.35%)	
Asian	3 (0.61%)	3 (0.77%)	0 (0%)	
Gender				0.77
Female	215 (43.17%	167 (42.71%)	48 (44.86%)	
Male	283 (56.83%)	224 (57.29%)	59 (55.14%)	
Cancer site				0.09
Bladder	9 (1.80%)	5 (1.27%)	4 (3.70%)	
Brain	19 (3.79%)	18 (4.58%)	1 (0.93%)	
Breast	8 (1.60%)	7 (1.78%)	1 (0.93%)	
GI	62 (12.38%)	53 (13.49%)	9 (8.33%)	
GU	31 (6.19%)	23 (5.85%)	8 (7.41%)	
Gynecologic	5 (0.99%)	5 (1.27%)		
Head and Neck	45 (8.98%)	36 (9.16%)	9 (8.33%)	
Liver	5 (0.99%)	2 (0.51%)	3 (2.78%)	
Lung	70 (13.97%)	53 (13.49%)	17 (15.74%)	
Lymphoma	5 (0.99%)	2 (0.51%)	3 (2.78%)	
Melanoma	126 (25.15%)	100 (24.45%)	26 (24.07%)	
Pancreatic	32 (2.79%)	24 (6.11%)	8 (7.41%)	
Rectal	3 (0.60%)	2 (0.51%)	1 (0.93%)	
Renal	18 (3.60%)	16 (4.07%)	2 (1.85%)	
Sarcoma	31 (6.19%)	25 (6.36%)	6 (5.56%)	
Skin (non-melanoma)	17 (3.40%)	12 (3.05%)	5 (4.63%)	
Other	15 (2.80%)	10 (2.54%)	5 (4.63%)	
Stage				0.80
I	11 (2.53%)	9 (2.56%)	2 (2.38%)	
II	45 (10.34%)	37 (10.54%)	8 (9.52%)	
III	71 (16.62%)	60 (17.09%)	11 (13.10%)	
IV	308 (70.80%)	245 (69.80%)	63 (75.00%)	
Distance from cancer center (zip code based) miles	38.16 (107.44)	40.95 (119.98)	28.02 (33.06)	0.06
Comorbidities				
HTN				
* Yes*	225 (44.91%)	169 (57.00%)	56 (48.15%)	0.13
* No*	276 (50.09%)	224 (43.00%)	52 (51.85%)	
CVD				
* Yes*	57 (11.38%)	41 (89.57%)	352 (85.19%)	0.27
* No*	444 (88.62%)	16 (10.43%)	92 (14.81%)	
HLD				
* Yes*	138 (37.54%)	110 (27.99%)	28 (25.93%)	0.76
* No*	363 (72.46%)	283 (72.01%)	80 (74.07%)	
T2DM				
* Yes*	78 (15.57%)	59 (15.01%)	334 (17.59%)	0.61
* No*	423 (84.43%)	19 (84.99%)	89 (82.41%)	
Obesity				
* Yes*	150 (29.94%)	124 (31.55%)	26 (24.07%)	0.17
* No*	351 (70.06%)	269 (68.45%)	82 (75.93%)	

Table 2 presents the results of the EXCEEDS triage process overall, as well as by the final category in which the patient was placed. Of the 391 respondents, 25% reported doing aerobic activity three times in the past week and 14% reported doing muscle-strengthening activities in the past week. In this group of respondents, 36% reported being highly confident they would exercise regularly without support from an exercise professional. Exercise confidence was associated with EXCEEDS triage category, with greater confidence among those whose answers indicated appropriate referral to community-based exercise as compared to those who reported symptoms consistent with a referral to rehabilitation services (*χ*^2^ = 42.96, *p* < 0.0001). There was a similar significant association between engaging in resistance exercise (yes/no) and EXCEEDS category (*χ*^2^ = 12.93, *p* = 0.002). There was also a significant association between engagement in aerobic exercise (yes/no) and EXCEEDS, such that those with fewer impairments reported greater confidence (*χ*^2^ = 32.35, *p* < 0.0001).

**Table 2:** EXCEEDS responses (*N* = 391)

	Overall	Rehabilitation services	Clinical supervised exercise	Community-based exercise oncology
	391	210	73	109
Do any of the following limit your ability to complete daily activities including work, hobbies, home care, socializing, and caring for yourself or loved ones?				
Difficulty getting in or out of a vehicle, or using public transportation	22	22	0	0
Difficulty walking one block without using a mobility aid like a cane or a walker	97	97	0	0
Difficulty moving or reaching with your arms	40	40	0	0
Difficulty being without a caregiver or another	26	26	0	0
Moderate to high pain that you rate as 7 out of 10 (0 = no pain, 10 = highest pain)	56	56	0	0
Moderate to high fatigue that you rate as 7 out of 10 (0 = no fatigue, 10 = highest fatigue)	78	78	0	0
New or worsening muscle weakness or coordination	60	60	0	0
Difficulty with eating or drinking	15	15	0	0
Difficulty with memory, multitasking, or thinking	15	15	0	0
Numbness or loss of sensation in feet or hands	38	38	0	0
Dizziness or blurred vision, or feeling lightheaded or disoriented	13	13	0	0
None of the Above	182	0	73	109
Have you fallen in the past 6 months?				
Yes	36`	30	6	0
No	355	180	67	109
In the past 3 months, have you had any of the following cancer treatments?				
Surgery	51	33	11	7
Stem cell transplant	3	1	0	2
Chemotherapy	186	116	41	29
Targeted therapy or immunotherapy	206	94	39	73
Radiation therapy or brachytherapy	50	33	10	7
None of the Above	20	10	2	8
In the past week, have you experienced any of the following conditions?				
Mild daily fatigue that you rate as 3 to 6 out of 10 (0 = no fatigue, 10 = highest fatigue	148	99	49	0
Tingling sensation or loss of feeling in your feet or hands	88	59	24	0
Difficulty with memory, multitasking, or thinking	42	32	10	0
Dizziness after you stand up from either sitting in a chair or from lying down	34	24	10	0
Nausea or frequent vomiting/diarrhea that interferes with daily activities	22	16	6	0
Frequent feelings of dehydration	8	5	3	0
Loss or gain of more than 5% body weight	25	21	4	0
Lack of voluntary control over urination or defecation (incontinence)	7	5	2	0
Persistent heaviness or swelling in your arm(s), leg(s), or trunk (lymphedema)	13	11	2	0
Immunosuppression due to anemia, neutropenia, or thrombocytopenia	6	5	1	0
None of the Above	168	55	4	109
Do you currently have or are you planning to receive a peripherally Inserted Central Cather (PICC), port, intraperitoneal catheter, or ostomy?				
Yes	150	102	22	26
No	242	108	51	83
During the past week have you performed 3 or more days of physical activity where your heart beats faster and your breathing is harder than normal for 30 min or more?				
Yes	97	35	13	49
No	204	140	23	41
During the past week have you performed 2 or more days of physical activity to increase muscle strength, such as lifting weights?				
Yes	53	21	6	26
No	247	154	30	63
Would you describe yourself as “highly confident” that you will exercise at moderate or vigorous intensity for 30 min, at least 3 times a week, for the next 12 weeks without receiving support from an exercise professional?				
Yes	142	47	35	60
No	250	163	38	49

## Discussion

Recent ASCO guidelines indicate that medical oncologists should refer patients to exercise to address symptoms and side effects [1]. A major challenge to achieving this goal lies in the need to triage patients to the appropriate kind of exercise or rehabilitation programming [6]. This process requires time and expertise that may be beyond the scope of medical oncologists. Oncologists self-report that they agree that patients should be referred to appropriate exercise programming, but that they do not have the time or training to do so [20]. As a result, 15% of patients report recalling being referred to an exercise program by their oncologist [4]. Systems are needed that include the buy-in of oncologists but require minimal time and effort. We implemented a clinical workflow to address this cancer care delivery challenge. This system is acceptable to clinicians (100% of medical assistants participated) and patients (78% of infusion patients completed the triage survey). The system appears to be feasible as well, given that 45% the patients who completed the triage survey and who were offered an intervention accepted the offer. The success of this approach, from the perspective of patients, nursing leadership, and oncology clinicians, suggests that there may be value to broader implementation of this approach at other cancer centers.

Implementation of this triage and referral process was accomplished using well-documented approaches suggested by a recent review of exercise oncology implementation [12] and followed the EPIS framework [11]. Buy-in from clinical leadership, including physicians and nurses, was crucial, as was informing the staff, adaptability with regard to fitting into the clinical flow, and ensuring that all medical assistant staff were comfortable with the new processes before starting. Audit and feedback processes enabled nursing management to understand which medical assistants needed to be reminded of the new procedures and resulted in 85% of infusion patients being offered the triage instrument. Acceptability is underscored by the seamless expansion of the program to an additional floor of Hillman Cancer Center after 5 months of the QI project being in operation.

We observed that a low percentage of patients triaged to community-based exercise accepted any referrals (15%). One hypothesized explanation for this would be that these patients did not value the referral because they saw no problem to be fixed. The rate of acceptance for the other two categories is consistent with this hypothesis. A comparatively higher percentage of patients triaged to clinically supervised exercise accepted the referral (35%). Further, 62% of patients triaged to rehabilitation accepted either a referral to rehabilitation services or clinically supervised exercise. That said, only 23.3% of patients triaged to rehabilitative services accepted the referral. The reasons for refusing the referral included cost, time, distance, prior negative experiences with physical therapy, or unwillingness to add additional appointments while going through chemotherapy. The patients who refused rehabilitation services were offered onsite clinically supervised exercise and 48.4% accepted this referral. Hypotheses for why the patient acceptance rate was so much higher for the onsite clinically supervised exercise include the co-location of the program, the lack of cost (the program is provided to patients without cost), and the convenience regarding timing with infusion visits. The high acceptance of this new clinical workflow by the clinicians and clinical staff is hypothesized to be the result of our facilitation efforts, coalition building activities, technical support, and adaptability to the clinical workflow. These observations may be useful for others interested in implementing similar triage programs with regards to the proportion of patients likely to be referred to community-based programming, clinically supervised exercise, and rehabilitation services. It also validates our prior published documentation of the value of co-location of exercise oncology and rehabilitation services with infusion therapy [21].

One prior study used a shorter “Ask Advise Refer” approach to triage and referral of 1174 patients [22]. Patients identified as being inadequately active were further evaluated to discern whether their performance status suggested value of a referral to rehabilitation services (physical or occupational therapy). A total of 540 patients were evaluated for performance status, 168 were referred to rehabilitation services. Of these, 13 (8%) accepted and completed the referral. Multiple differences between this prior approach and the current approach explain differences. First, My Wellness Check was integrated into the electronic medical record. We are working toward this goal at Hillman Cancer Center. Further, unlike our Hillman Cancer Center program, the My Wellness Check project did not make any referrals to exercise programming. Finally, unlike the My Wellness Check project, we integrated a rehabilitation navigator to talk the patients through the results of the triage tool and personally explain the referral.

Limitations of our study include that we were unable to follow-up on the 49 patients who accepted referral to rehabilitation services to discern whether they were connected to those services because services outside of our health system were chosen. Further, the focus of this project was to evaluate implementation of triage and referral, and therefore, we are unable to comment on the effectiveness of the exercise and rehabilitation interventions.

We have documented high acceptability and feasibility of the implementation of a new clinical workflow into a busy academic cancer center, in two chemotherapy infusion clinics, as well as excellent implementation of the triage process by medical staff (85%), acceptability of the triage process by patients (78%) and feasibility of referrals (45%). This process holds promise as an approach to making exercise oncology and rehabilitation referrals standard of practice in the setting of medical oncology. Making exercise and rehabilitation triage and referral standard of care will assist with addressing documented disparities in physical function, quality of life, and mental health, and perhaps survival, by race, ethnicity, geography (urban versus rural), and socioeconomic status [23–26].

## References

[1] Ligibel JA, Bohlke K, May AM et al (2022) Exercise, diet, and weight management during cancer treatment: ASCO Guideline. J Clin Oncol 40(22):2491–2507. 10.1200/JCO.22.00687 35576506

[2] Rock CL, Thomson CA, Sullivan KR et al (2022) American Cancer Society nutrition and physical activity guideline for cancer survivors. CA Cancer J Clin 72(3):230–262. 10.3322/caac.21719 35294043

[3] Campbell KL, Winters-Stone KM, Wiskemann J et al (2019) Exercise guidelines for cancer survivors: consensus statement from International Multidisciplinary Roundtable. Med Sci Sports Exerc 51(11):2375–2390. 10.1249/MSS.0000000000002116 PMC8576825 31626055

[4] Ligibel JA, Pierce LJ, Bender CM et al (2022) Attention to diet, exercise, and weight in oncology care: results of an American Society of Clinical Oncology national patient survey. Cancer 128(14):2817–2825. 10.1002/cncr.34231 35442532

[5] Jones LW, KS C, Fairey AS, Mackey JR (2004) Effects of an oncologistʼs recommendation to exercise on self-reported exercise behavior in newly diagnosed breast cancer survivors: a single-blind, randomized controlled trial. Ann Behav Med 28(2):105–113 10.1207/s15324796abm2802_5 15454357

[6] Stout NL, Brown JC, Schwartz AL et al (2020) An exercise oncology clinical pathway: screening and referral for personalized interventions. Cancer 126(12):2750–2758. 10.1002/cncr.32860 PMC7258139 32212338

[7] Schmitz KH, Campbell AM, Stuiver MM et al (2019) Exercise is medicine in oncology: engaging clinicians to help patients move through cancer. CA Cancer J Clin 69(6):468–484. 10.3322/caac.21579 PMC7896280 31617590

[8] Mitchell SA, Chambers DA (2017) Leveraging implementation science to improve cancer care delivery and patient outcomes. J Oncol Pract 13(8):523–529. 10.1200/JOP.2017.024729 PMC5555033 28692331

[9] Covington KR, Marshall T, Campbell G et al (2021) Development of the Exercise in Cancer Evaluation and Decision Support (EXCEEDS) algorithm. Support Care Cancer 29(11):6469–6480. 10.1007/s00520-021-06208-7 PMC8525648 33900458

[10] Covington KR MT, Sharp J, Kendig T, Williams G, Pergolotti M. (2021) Utility and acceptability of the EXCEEDS algorithm for oncology stakeholders: results of a Delphi study. J Clin Oncol ;39(15):abstract e13538. 10.1200/JCO.2021.39.15_suppl.e13538

[11] Aarons GA, Hurlburt M, Horwitz SM (2011) Advancing a conceptual model of evidence-based practice implementation in public service sectors. Adm Policy Ment Health 38(1):4–23. 10.1007/s10488-010-0327-7 PMC3025110 21197565

[12] Adsul P, Schmitz K, Basen-Engquist KM, Rogers LQ (2022) Studying the implementation of exercise oncology interventions: a path forward. Transl J Am Coll Sports Med Fall 7(4):1–8. 10.1249/tjx.0000000000000208 PMC9674226 36407802

[13] Ogrinc G, Davies L, Goodman D, Batalden P, Davidoff F, Stevens D (2015) Squire 2.0 (Standards for Quality Improvement Reporting Excellence): revised publication guidelines from a detailed consensus process. Am J Crit Care 24(6):466–73. 10.4037/ajcc2015455 26523003

[14] Leeman J, Rohweder C, Lee M, et al (2021) Aligning implementation science with improvement practice: a call to action. Implement Sci Commun 8 2(1):99. 10.1186/s43058-021-00201-1 PMC8424169 34496978

[15] Harris PA, Taylor R, Minor BL et al (2019) The REDCap consortium: building an international community of software platform partners. J Biomed Inform 95:103208. 10.1016/j.jbi.2019.103208 PMC7254481 31078660

[16] Harris PA, Taylor R, Thielke R, Payne J, Gonzalez N, Conde JG (2009) Research electronic data capture (REDCap)–a metadata-driven methodology and workflow process for providing translational research informatics support. J Biomed Inform 42(2):377–381. 10.1016/j.jbi.2008.08.010 PMC2700030 18929686

[17] Huang HP, Wen FH, Yang TY et al (2019) The effect of a 12-week home-based walking program on reducing fatigue in women with breast cancer undergoing chemotherapy: a randomized controlled study. Int J Nurs Stud 99:103376. 10.1016/j.ijnurstu.2019.06.007 31442785

[18] Loh KP, Sanapala C, Janelsins M et al (2022) Protocol for a pilot randomized controlled trial of a mobile health exercise intervention for older patients with myeloid neoplasms (GO-EXCAP 2). J Geriatr Oncol 13(4):545–553. 10.1016/j.jgo.2021.12.011 PMC9058183 34949540

[19] Batalik L, Winnige P, Dosbaba F, Vlazna D, Janikova A (2021) Home-based aerobic and resistance exercise interventions in cancer patients and survivors: a systematic review. Cancers (Basel) 13(8):1915. 10.3390/cancers13081915 PMC8071485 33921141

[20] Ligibel JA, Jones LW, Brewster AM et al (2019) Oncologistsʼ attitudes and practice of addressing diet, physical activity, and weight management with patients with cancer: findings of an ASCO survey of the oncology workforce. J Oncol Pract 15(6):e520–e528. 10.1200/JOP.19.00124 PMC6827390 31095436

[21] Schmitz KH, Potiaumpai M, Schleicher EA et al (2021) The exercise in all chemotherapy trial. Cancer 127(9):1507–1516. 10.1002/cncr.33390 33332587

[22] Brick R, Natori A, Moreno PI, Molinares D, Koru-Sengul T, Penedo FJ (2023) Predictors of cancer rehabilitation medicine referral and utilization based on the Moving Through Cancer physical activity screening assessment. Support Care Cancer 31(4):216. 10.1007/s00520-023-07679-6 PMC10281047 36928440

[23] Bulls HW, Chang PH, Brownstein NC et al (2022) Patient-reported symptom burden in routine oncology care: examining racial and ethnic disparities. Cancer Rep (Hoboken) 5(3):e1478. 10.1002/cnr2.1478 PMC8955049 34165256

[24] Lloyd-Williams M, Shiels C, Dowrick C, Kissane D (2021) Socio-economic deprivation and symptom burden in UK Hospice patients with advanced cancer-findings from a longitudinal study. Cancers (Basel) 13(11):2537. 10.3390/cancers13112537 PMC8196745 34064172

[25] Blake KD, Moss JL, Gaysynsky A, Srinivasan S, Croyle RT (2017) Making the case for investment in rural cancer control: an analysis of rural cancer incidence, mortality, and funding trends. Cancer Epidemiol Biomarkers Prev 26(7):992–997. 10.1158/1055-9965.EPI-17-0092 PMC5500425 28600296

[26] Anderson T, Herrera D, Mireku F et al (2023) Geographical variation in social determinants of female breast cancer mortality across US counties. JAMA Netw Open. 6(9):e2333618. 10.1001/jamanetworkopen.2023.33618 PMC10502521 37707814

